# Increased Lytic Efficiency of Bovine Macrophages Trained with Killed Mycobacteria

**DOI:** 10.1371/journal.pone.0165607

**Published:** 2016-11-07

**Authors:** Ramon A. Juste, Marta Alonso-Hearn, Joseba M. Garrido, Naiara Abendaño, Iker A. Sevilla, Christian Gortazar, José de la Fuente, Lucas Dominguez

**Affiliations:** 1 Animal Health Department, NEIKER-Tecnalia, Berreaga, 1, 48160 Derio, Bizkaia, Spain; 2 SaBio, Instituto de Investigación en Recursos Cinegéticos, IREC (CSIC, UCLM, JCCM), Ronda de Toledo, 13071, Ciudad Real, Spain; 3 Department of Veterinary Pathobiology, Center for Veterinary Health Sciences, Oklahoma State University, 250 McElroy Hall, Stillwater, OK, 74078, United States of America; 4 Centro de Vigilancia Sanitaria Veterinaria (VISAVET), Universidad Complutense, Avenida Puerta de Hierro, 28040, Madrid, Spain; University of Delhi—South Campus, INDIA

## Abstract

Innate immunity is evolutionarily conserved in multicellular organisms and was considered to lack memory until very recently. One of its more characteristic mechanisms is phagocytosis, the ability of cells to engulf, process and eventually destroy any injuring agent. We report the results of an *ex vivo* experiment in bovine macrophages in which improved clearance of *Mycobacterium bovis* (*M*. *bovis*) was induced by pre-exposure to a heat killed *M*. *bovis* preparation. The effects were independent of humoral and cellular adaptive immune responses and lasted up to six months. Specifically, our results demonstrate the existence of a training effect in the lytic phase of phagocytosis that can be activated by killed mycobacteria, thus suggesting a new mechanism of vaccine protection. These findings are compatible with the recently proposed concept of trained immunity, which was developed to explain the observation that innate immune responses provide unspecific protection against pathogens including other than those that originally triggered the immune response.

## Introduction

Innate immunity is evolutionarily conserved in multicellular organisms and is the most primitive component of the immune system. It is based on non-specific mechanisms that, in contrast to the adaptive immune response, were considered to lack memory until very recently. The traditional paradigm in immunology is that innate immunity—as opposed to adaptive immunity—lacks of memory-like properties. In plants and invertebrates, however, memory effect for innate host defence has been recently recognized and defined as training to differentiate from specific immune memory. Therefore, the term 'trained immunity' was proposed to describe the potentiating effects of an exposure to microbial agents or vaccines on innate immune responses, which would thus mount a more effective immune response against related and sometimes even unrelated infections. This training leads to increased cytokine production via epigenetic reprogramming of monocytes [1]. Although the epidemiological evidence of Bacille Calmette-Guérin (BCG) vaccine effectiveness against tuberculosis is referred to as proof of the trained immunity concept in higher animals [2], no experimental evidence has been produced to support the hypothesis that phagocytosis might be playing a critical role in this process.

Here, we report an improved clearance of *Mycobacterium bovis* in bovine macrophages lasting up to six months and independent of specific immune responses. To discriminate between the adaptive and innate immune responses in cattle sensitized with a killed *M*. *bovis* preparation, monocyte derived macrophages (MDM) from sensitized and control cattle were tested *ex vivo* for phagocytic activity while monitoring for systemic specific immune responses. Our results demonstrate the existence of a training effect in the lytic phase of phagocytosis that can be activated by killed mycobacterial cells. This suggests a new mechanism of immune protection by which low-intensity priming enhances early phagocytic responses independently of humoral and cellular adaptive immune responses for, at least, six months. This mechanism would support the empirical evidence of protection induced by killed mycobacterial vaccines [3,4,5]. These findings fit well with the recently proposed general concept of trained immunity, which was developed to explain the observation that innate immune responses provide unspecific protection against pathogens including other than those that originally triggered the immune response.

## Materials and Methods

### Ethics statement

Animals used in this study were submitted only to procedures that according to European (Directive 2010/63/EU of the European Parliament and of the Council of 22 September 2010 on the protection of animals used for scientific purposes. Chapter 1, Article 1, Section 5, paragraphs b and f) and Spanish (Real Decreto 53/2013, de 1 de febrero, por el que se establecen las normas básicas aplicables para la protección de los animales utilizados en experimentación y otros fines científicos, incluyendo la docencia, Article 2, Section 5, Paragraphs b and f) legislation on experimental animals are exempt from its application. The animals, belonging to a registered commercial farm supervised by the local livestock authority (Servicio de Ganadería de la Diputación Foral de Bizkaia) were submitted only to the introduction of a needle in accordance with good veterinary practice and were not killed in relationship with this study.

### Animals and treatment

Twenty-five 3- to 6-month-old Limousin (6), Pyrenean (8) or crossed (11) cattle originally purchased from 8 different farms and currently housed in a small commercial feedlot were intramuscularly injected with 2 ml of a suspension of heat-inactivated cells of a local *M*. *bovis* isolate (NEIKER MS#1403) in Montanide 50 (SEPPIC, Paris, France) (MdR) or left untreated. Ten animals received a full dose of 10^7^ colony forming units (CFU) (FD group), ten a reduced dose of 10^3^ CFU (RD group), and 5 animals served as untreated controls (UC group). All breeds were present in each treatment group. Two animals from the FD group, 3 from the RD group and 2 from the UC group were lost because of unrelated disease (1), traumatism (1) or sale (5) before the final test.

### Humoral immune response: ELISA test

On day 0, immediately before treatment, and at 15, 43, 78 and 186 days post-treatment (dpt), blood was obtained from each animal via the tail vessels and collected in EDTA Vacutainer tubes (Becton, Dickinson and Company, Sparks, MD, USA). The blood samples were analysed using an in-house anti-*M*. *bovis* ELISA with bovine PPD as the antigen [5]. This assay includes a serum *Mycobacterium phlei* pre-absorption step to reduce non-specific reactivity caused by environmental mycobacteria. The results of this test were expressed as relative optical density (OD) calculated as raw serum OD divided by the OD value of the positive control.

### IFN-γ release assay (IGRA)

Immediately before treatment and at 15, 43, 78 and 186 dpt blood stimulation was performed within the first 8 h after sampling in 24-well culture plates (Becton-Dickinson®, Franklin Lakes, NJ) with either phosphate-buffered saline (PBS), avian purified protein derivative (PPDAV) (CZ Veterinaria® SA, Porriño, Spain) or bovine purified protein derivative (PPDBOV) (CZ Veterinaria® SA, Porriño, Spain) tuberculin. After incubation for 16–24 h at 37°C + 5–7% CO_2_, plasma was separated by centrifugation and frozen at -20°C until testing. Subsequently, a commercial IGRA (BovigamTM, Fisher Scientific, Schlieren, Switzerland) was performed in accordance with the manufacturer’s instructions.

### Intradermal reaction

At 46 and 81 dpt, each animal was intradermally inoculated at three spots on each side of the neck (ST) with 0.1 ml of standard bovine and avian tuberculin (CZV, SA. Porriño, Spain) or PBS using a Dermojet device (AKRA Dermojet, Pau, France). Reactions were read after 72 h according to the Spanish TB control program regulations.

### Phagocytosis assay

The phagocytosis assays were performed at 78 and 186 dpt to estimate the bacterial load reduction associated with each treatment as previously described [6,7]. For the first phagocytic assay, peripheral blood was drawn into heparinized Vacutainer tubes, diluted 1:2 in Hanks balanced salt solution (HBSS), layered over 10 ml of Ficoll-Paque gradient (1.084 g/cm^3^) and centrifuged at 900 x g for 30 min. The cell interphase was collected and centrifuged at 400 x g for 10 min to remove platelets from peripheral blood monocytic cells (PBMCs). For the second test, macrophages were magnetically enriched from PBMCs using magnetic activated cell sorting (MACS) technology (Miltenyi Biotech, Bergisch Galdbach, Germany). In both cases, bovine monocytes were resuspended in RPMI-1640 supplemented with 20 mM L-glutamine, 10% heat-inactivated bovine serum, 100 U ml^-1^ penicillin G and 100 mg ml^-1^ streptomycin sulfate (Lonza) and seeded into 24-well plates at a density of 4 x 10^5^ cells ml^-1^. After 2 h at 37°C in a humidified 5% CO_2_ incubator, non-adherent cells were removed. Adherent cells were incubated for 7 days at 37°C to allow differentiation to MDM prior to infection. Differentiated MDMs were inoculated with a single-cell suspension of *M*. *bovis* at an MOI (multiplicity of infection; bacteria:cells) of 10:1. After 2 h and 7 days, the supernatant was removed from three wells, and the cells were washed twice with HBSS to remove extracellular bacteria. Infected macrophages were lysed by vigorous pipetting with 0.5 ml of 0.1% Triton X-100 (Sigma-Aldrich) in sterile water for 10 min. Supplemented Mycobacteria Growth indicator tubes (MGIT) (Becton, Dickinson and Company, Sparks, MD) were inoculated with 0.1 ml of each initial bacterial suspension and with the 2 h and 7 d p. i. cell lysates. The tubes were incubated at 37 ± 2°C for up to 41 days in a Bactec MGIT 960 instrument (Becton, Dickinson and Company). The earliest instrumental indication of positivity (i.e., time to detection [TTD]) for each tube was recorded. The predicted number of bacteria in each positive tube was calculated using previously generated mathematical formulas which relate TTD (in days) to estimated log_10_ CFUs. Bacterial cell reduction between 2 h and 7 days p.i. were calculated by dividing the estimated log_10_ CFUs at day 7 by that at 2 h p.i.

### Cytokines gene expression

At 186 dpt, MDMs purified from peripheral blood of treated and untreated animals were infected *ex vivo* with *M*. *bovis*. At 24 h pi, the infected MDM were washed in 0.5 ml of cold HBSB, mixed with 50 μl of Lysis Solution and incubated at room temperature for five minutes to allow RNA release into the Lysis solution (PowerSYBR® Green Cells-to-Ct kit (Life Technologies, Carlsbad, CA). DNAse I was added to the Lysis Solution to allow genomic DNA degradation at this step. Next, 5 μl of Stop Solution were mixed into the lysate to inactivate the lysis solution so that it would not interfere with the reverse transcription (RT) or polymerase chain reaction (PCR). Cell lysates (10 μl) were then reverse transcribed to synthesize cDNA using 2.5 μl of 20 X RT Enzyme Mix, 25 μl of 2X SYBR RT Buffer, and 12.5 μl of Nuclease-free water. The reaction mixtures were incubated at 37°C for 60 min and then at 95°C for 5 min to inactivate the RT enzyme. Finally, the synthesized cDNAs were amplified by real-time PCR using Power SYBR Green PCR Master Mix and PCR primers for the bovine IL1-α, TGF-β, BCL2, TNF-α and C3 genes as previously described [6]. The β-actin gene was used as the endogenous control gene in the assays. To determine the changes in gene expression (Relative quantification, RQ), the following formula was used: RQ = 2-Δ(ΔC_T_) were ΔC_T_ is C_T_ (target gene)—C_T_ (β-actin) and Δ(ΔC_T_) is ΔC_T_ (experimental)—ΔC_T_ (control). Results were expressed as RQ of transcription compared to those of control uninfected cells.

### Statistical analysis

ELISA and IGRA optical density readings, estimated bacterial counts, reductions in bacterial load and cytokine expression estimates were compared using the GLM procedure in the SAS 9.1 software (SAS Institute, Cary, NC) with the Tukey-Kramer multiple-comparison post-test. Correlations between variables to explore their association with innate or adaptive responses were examined by the Kendall correlation and principal component analysis with SAS software. In all predictive analyses, differences were considered statisticallly significant at p<0.05.

## Results

### Assessment of humoral and cell-mediated immune responses in cattle treated with a heat killed *M bovis* preparation

**Fig 1:**
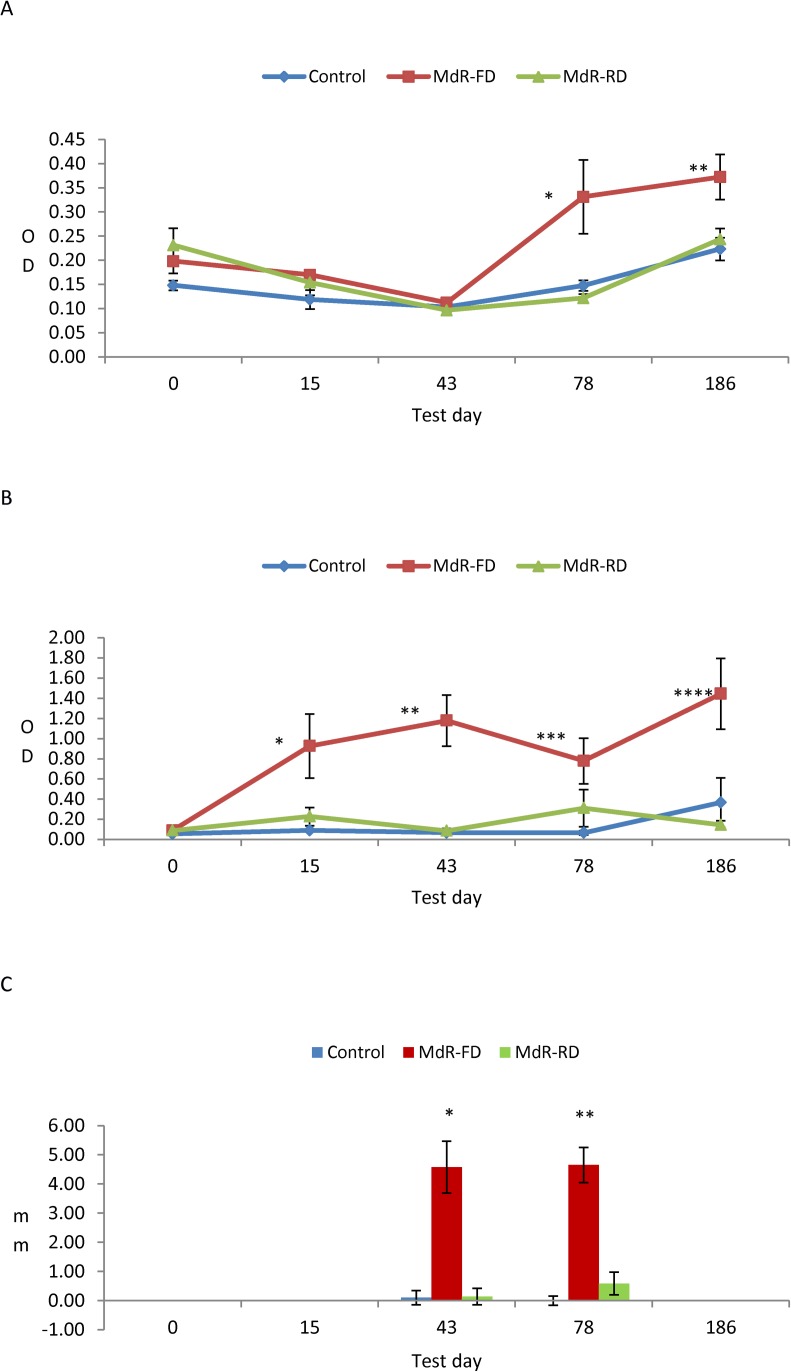
Immune dynamics throughout the experiment. (A) ELISA: Specific humoral immune response in relative optical density units in TB ELISA. * Comparison between MdR-FD and Control (p = 0.0007) and MdR-RD (<0.0001) groups at day 78. ** Comparison between MdR-FD and Control (p = 0.0239) and MdR-RD (p<0.0110) groups at day 186. Other days differences are not statistically significant at p<0.0500. (B) IFN: Specific cellular immune response in optical density units in Interferon γ release assay. * Comparison between MdR-FD and Control (p = 0.0061) and MdR-RD (p = 0.0051) groups at day 15. ** Comparison between MdR-FD and Control (p = 0.0003) and MdR-RD (<0.0110) groups at day 43. *** Comparison between MdR-FD and Control (p = 0.0190) and MdR-RD (p = 0.0580) groups at day 78. **** Comparison between MdR-FD and Control (p = 0.0043) and MdR-RD (p<0.0001) groups at day 186. Other days differences are not statistically significant at p<0.0500. (C) Skin test: Mean group skin test results in mm. * Comparison between Control and MdR-FD (p<0.0001) or MdR-RD (p = 0.9678) and between both MdR groups (p<0.0001) at day 43. ** Comparison between Control and MdR-FD (p<0.0001) or MdR-RD (p = 0.5456) and between both both MdR groups (p<0.0001) at day 78. Control: Untreated control group. MdR-FD: Full-dose group. MdR-RD: Reduced-dose group.

For adaptive immunity, three standard tests based on humoral and cell-mediated immune responses were conducted to determine: i) antigen-specific antibody production, ii) IFN-γ release by peripheral blood lymphocytes and iii) cell infiltration into the skin [8]. None of the treated groups exhibited humoral responses at 0, 15 and 43 dpt) (Fig 1A). However, the group treated with the highest dose of the killed *M*. *bovis* preparation displayed a significant increase in antibody levels by day 78 that were slightly higher by day 186. The control and low-dose groups on day 186 also exhibited slight increases in antibody levels relative to their minimal readings, but the antibody levels did not differ among these groups. These patterns are consistent with the anamnestic effect of the tuberculin antigens used in the skin test, which increases the reactivity of infected anergic animals [9]. The cellular response as measured by IFN-γ release assay (IGRA) by peripheral blood lymphocytes revealed a substantial specific response by 15 dpt in the FD group that was significantly different from that of the control group (Fig 1B). However, both the control and low-dose immune responses converged at a very low level on day 43 when the immune response of the FD group reached a maximum.

Regarding the intradermal test, at the first test only the FD group exhibited a significant increase in mean skin thickness, whereas the control and low-dose RD groups exhibited negligible increases in skin thickness. By the second test, the high-dose group remained at a similar reactivity level, whereas reactivity remained very low in both the control and RD groups. With the exception of the low-level response of the RD group, these specific immune response dynamics were consistent with expectations.

### *M*. *bovis* phagocytosis assessment in bovine macrophages purified from blood of treated and untreated cattle

**Fig 2:**
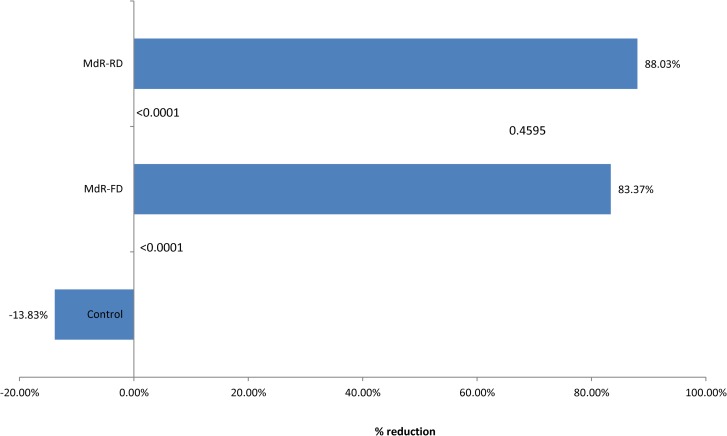
Average mycobacterial load reduction according to the treatment calculated as the quotient between log_10_ CFU at 2 h minus log_10_ CFU at 7 days and log_10_ CFU at 2 h. The four decimal numbers in the base of the bars are the p values for differences between the control and sensitized groups. The number between MdR-FD and MdR-RD at the end of the bars represent the p value for the difference between sensitized groups. Control: Untreated control group. MdR-FD: Full-dose group. MdR-RD: Reduced-dose group.

At 78 and 186 dpt, MDM purified from peripheral blood of treated and untreated animals were infected *ex vivo* with *M*. *bovis*. Bacterial load associated with each treatment at 2 h and 7 days p. i. was estimated as previously described [6,7]. In the phagocytosis test, bacterial cell uptake did not differ among the groups (S2 Table). Fig 2 summarises the results of the two phagocytosis assays for average bacterial load reduction at 7 days p. i. The FD group exhibited an average reduction of 83.37%, whereas the RD group displayed a reduction of 88.03%. By contrast, the bacterial load increased slightly in the control group. Notably, in the phagocytosis assay performed at 78 dpt, the control group exhibited an approximately 50% reduction that made the reductions in the treated groups appear less dramatic by comparison over time (Table 1). This response could be a short-term effect caused by intradermal sensitization with tuberculin or it might be related to individual variation. Interestingly, the rate of infection clearance was much higher in this sampling than in the sampling conducted three months after the last skin test (186 dpt). This smaller but more widespread reduction together with the variable reductions shortly after skin sensitization or boosting and three months later suggest that phagocytic memory might not be constant and definitive but vary with time and tend to wane after several months. This effect of the treatment on the macrophages lytic capability has not been described previously and may have important implications for explaining natural and vaccine protection mechanisms during chronic intracellular infections.

**Table 1:** Results of the phagocytosis assays.

		MD78				MD186			
Treatment	Calf code	Uptake	7 days	Reduction		Uptake	7 days	Reduction	
				Time	Treatment			Time	Treatment
Full Dose	1656 C	6.0183	0.0000	100%	• 83%^a^ • 63%^b^ • 83%^c^	.	.	.	• 84%^a^ • 78%^b^ • 14%^c^
	1657 C	.	.	.		.	.	.	
	3621 L	.	.	.		3.2502	0.7869	76%	
	4159 C	.	.	.		3.9288	0.5586	86%	
	4166 C	5.9311	0.0000	100%		4.5379	0.0000	100%	
	4168 C	6.4247	6.3932	0%		3.9444	0.7647	81%	
	4170 C	.	.	.		.	.	.	
	6314 P	6.4535	0.0000	100%		5.2150	0.6587	87%	
	6315 P	6.2336	0.0000	100%		3.6865	0.9331	75%	
	6568 C	6.8783	0.0000	100%		3.7228	0.7680	79%	
Reduced Dose	0723 L	5.6854	0.0000	100%	• 95%^a^ • 88%^b^ • 67%^c^	4.3161	0.6490	85%	• 89%^a^ • 89%^b^ • 16%^c^
	1654 L	.	.	.		.	.	.	
	1655 P	.	.	.		.	.	.	
	4160 L	.	.	.		4.5011	0.8700	81%	
	4163 C	6.5538	1.3163	80%		6.1269	0.6506	89%	
	4164 L	6.7075	0.0000	100%		5.5988	0.0000	100%	
	4167 C	.	.	.		5.1879	0.5797	89%	
	5049 P	6.6816	0.0000	100%		4.5457	0.7110	84%	
	6310 P	6.4247	0.7983	88%		.	.	.	
	9681 P	6.7596	0.0000	100%		.	.	.	
Control	1658 C	6.4247	0.0000	100%	• 56%^a^ • 0%^b^ • 50%^c^	.	.	.	• -15%^a^ • 0%^b^ • 0%^c^
	3877 C	.	.	.		.	.	.	
	4165 L	6.3833	5.4100	15%		4.7049	6.0817	-29%	
	6312 P	6.3428	6.0872	4%		5.3117	5.9606	-12%	
	9682 P	6.8916	0.0000	100%		3.6748	3.6748	0%	

Individual bacterial cell reduction (Time) between 2 h and 7 days p.i. were calculated by dividing the difference between the estimated log_10_ CFUs at day 7 and that at 2 h p.i. by the log_10_ CFU at 2 h p.i. Group bacterial reduction was calculated as follows

^a^ Mean log_10_ CFU reduction from uptake to end of culture at 7 days p.i.

^b^ Reduction in mean log_10_ CFU in the treated group compared with the control group

^c^ Proportion of individuals with 100% reduction in the bacterial load in macrophages.

C: Crossed; P: Pyrenean; L: Limousin.

### Assessment of cytokine profiles in MDMs purified from treated and untreated cattle and infected *ex vivo* with *M*. *bovis*

At 24 h pi, RNA was extracted from *M*. *bovis*-infected MDMs and reverse transcribed to cDNA to measure IL1-α, TGF-β, BCL2, TNF-α and C3 expression levels by using qRT-PCR (S2 Table). The cytokine profiles of the groups did not differ significantly, except the *C*3 levels, which were significantly higher in both treated groups when compared to controls (p = 0.0006; p = 0.0327). Furthermore, C3 expression levels were significantly higher in the FD group than the RD group (p = 0.0042). This finding is consistent with the previously reported increase in the expression of C3 in wild boar tissues treated with this inactivated *M*. *bovis*-based immunogen [5, 10]. In addition, the up-regulation of C3 in the lymph nodes and tonsils has been correlated with tuberculosis resistance in wild boar [11].

### Relationships between immunological variables

**Fig 3:**
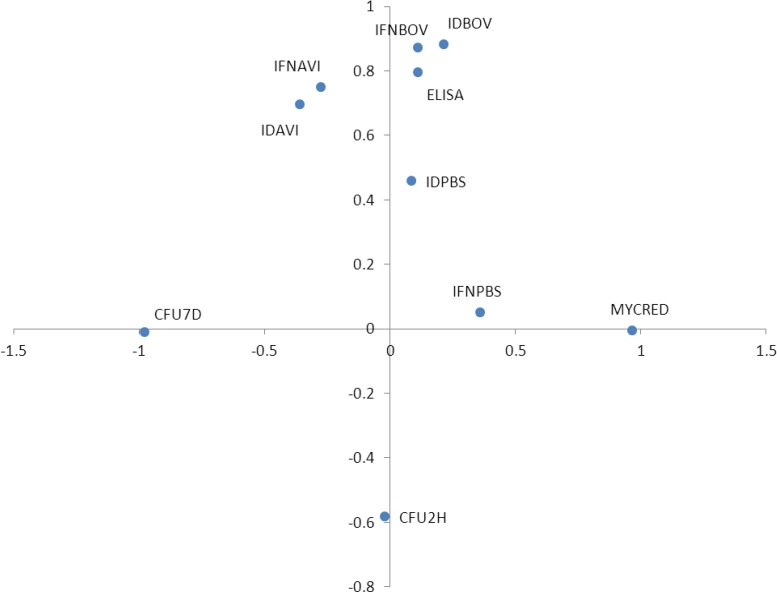
Principal component analysis of the relationships between variables. Note that bacterial uptake is negatively correlated with humoral and cellular specific immune responses. The bacterial reduction exhibits no correlation with these specific variables. Bacterial reduction defines the second factor, which is weakly correlated with the non-specific IFN response. IFNBOV: IFN with bovine PPD; IFNAVI: IFN with avian PPD; IFNPBS: IFN blank; IDBOV: intradermal test with bovine PPD; IDAVI: intradermal test with avian PPD; IDPBS: intradermal test blank; CFU2H: *M*. *bovis* CFU logarithm at 2 h after culture inoculation; CFU 7D: *M*. *bovis* CFU logarithm at 7 days after culture inoculation; MYCRED: *M*. *bovis* CFU reduction between 2 h and 7 days after inoculation.

Principal component analysis revealed two main associations between the studied variables (Fig 3). The first component represented the specific immune response because humoral and cellular specific response variables tended to cluster on the positive end. The second component represented bacterial lysis because the bacterial load at 7 days and load reduction clearly aligned along this axis. Strikingly, these two variables did not exhibit any correlation with the first component, suggesting that phagocytic activity decreases as specific mechanisms become dominant in the immune response. Therefore, phagocytosis becomes less relevant as more specific mechanisms are triggered. The lack of correlation between specific mechanisms and actual *M*. *bovis* clearance suggest that the innate component of the immune response is uncoupled from both specific responses and bacterial uptake.

## Discussion

Our results demonstrate that sensitizing young animals with an inactivated mycobacterial preparation by the parenteral route can boost the bacterial killing efficiency of infected macrophages. This is the first study to demonstrate a memory effect on phagocytic lysis in a higher mammal. This phenomenon differs from that reported in invertebrates, in which the learning effect appears to occur only at the uptake phase [12]. Because no cells other than macrophages were supposed to be present in our MDM infected cultures given that both variants of selective procedures applied yielded similar results, it could be assumed that the memory effect was maintained by macrophages themselves and not by lymphocytes interacting with them. Additionally, both the lack of correlation with specific cellular and humoral immune responses and the positive association with the non-specific IFN-γ response further demonstrate that phagocytosis does not depend on specific immune responses. Our results confirm that trained immunity is present in organisms other than mice and lower vertebrates at the macrophage level. Most importantly, our results indicate that this effect remains active in young adults for at least six months after exposure. Intriguingly, lower doses of killed-mycobacteria might be more efficient at inducing trained immune responses than higher doses, in agreement with field observations [3, 13, 14].

Mycobacteria are powerful immunogens, and this property has long been used to stimulate immune responses by adding mycobacteria immunogens to antigens of interest in complete Freund’s adjuvant [15]. This immunogenicity is consistent with the high stability of immune response-inducing genes, which is interpreted as an evolutionary adaptation favouring long-term survival in organs and subsequent dissemination [16, 17]. In addition, mycobacterial vaccines prevent other diseases non-specifically [2, 18, 19]. This study results suggest that the long held principle that tuberculosis killed vaccines do not work might not be true and that Tuberculosis vaccination could be achieved without using live bacilli, which pose a risk to immunocompromised individuals of any age who submit to BCG therapy, such as cancer patients. It is very important to define appropriate immunization protocols that can take advantage of the non-specific and powerful effect of killed vaccines on phagocytosis. These vaccination strategies may also provide protection against infections or immune dysfunctions other than *M*. *bovis*. Another interesting finding of this study, which unfortunately could not be extended, is that the training effect of macrophage phagocytosis persists for at least several months.

In summary, we present evidence that bovine macrophages can be “trained” to improve their lytic capacity, rather than just particle uptake, likely by epigenetic reprogramming [20, 21]. Our results are consistent with the concept of trained innate immunity recently proposed by Netea et al. [1]. Evidence from this study suggests that efficient priming of the phagocytic innate mechanisms can be induced by contact with bacterial structures present in killed slow-growing microbes. The challenge of faster-growing pathogens that can easily overwhelm the innate immune system would have prompted the development of an adaptive immune system that is not only able to address intracellular slower-growing microbes, but also requires larger amounts of stimuli to be triggered. Mastering the training of the innate mechanisms could cause a revolutionary shift in the preventive and therapeutic use of immunogens covering an extraordinarily broad range of diseases, from viral to chronic inflammatory and neoplastic diseases [22].

## Supporting Information

S1 Table Significant or nearly significant Pearson correlations between *M*. *bovis* uptake and reduction with other variables. Individual values at testing days 78 and 186 are shown.
(DOCX)

S2 Table Summary of results according to treatment and variable. MycRed: Least square (LS) mean and standard error of the mean (SEM) mycobacterial count reduction from 2 h post-inoculation to 7 days post-inoculation. ELISA186: LS mean and SEM antibody ELISA OD at 186 days post-inoculation. IFN186: LS mean and SEM interferon release assay at 186 days post-inoculation. ST81: LS mean and SEM skin test thickness increase at 81 days post-inoculation. IL1: LS mean and SEM interleukin 1 gene expression. TGFB: LS mean and SEM transforming growth factor beta gene expression. BCL2: LS mean and SEM bovine B-cell lymphoma/leukemia-2 apoptosis suppressant gene expression. TNF: LS mean and SEM tumour necrosis factor alpha gene expression. C3: LS mean and SEM complement 3 gene expression. Control: Untreated control group. MdR-FD: Killed *M*. *bovis* full-dose group. MdR-RD: Killed *M*. *bovis* reduced-dose group.
(DOCX)

## References

[1] Netea MG, Quintin J, van der Meeer JW. Trained immunity: A memory for innate host defense. Cell Host and Microbe 2011:9: 355–361. 10.1016/j.chom.2011.04.006 21575907

[2] Aaby P, Stabell-Benn C. Saving lives by training innate immunity with bacille Calmette-Guérin vaccine. Proc Natl Acad Sci USA 2012 109: 17317 10.1073/pnas.1215761109 23071307 PMC3491466

[3] Garrido JM, Vazquez P, Molina E, Plazaola JM, Sevilla IA, Geijo MV, et al Protection against tuberculosis in eurasian wild boar vaccinated with heat-inactivated Mycobacterium bovis. PLoS One 2011;6: e24905 10.1371/journal.pone.0024905 21935486 PMC3173485

[4] Alonso-Hearn M., Molina E, Geijo M, Vazquez P, Sevilla IA, Garrido JM, et al Immunization of adult dairy cattle with a new heat-killed vaccine is associated with longer productive life prior to cows being sent to slaughter with suspected paratuberculosis. J Dairy Sci 2012;95: p. 618–629. 10.3168/jds.2009-2860 22281327

[5] Beltrán-Beck B, De la Fuente J, Garrido JM, Aranaz A, Sevilla I, Villar M, et al Oral vaccination with heat inactivated Mycobacterium bovis activates the complement system to protect against tuberculosis. PLoS One 2014;9: e98048 10.1371/journal.pone.0098048 24842853 PMC4026474

[6] Abendaño N, Sevilla IA, Prieto JM, Garrido JM, Juste RA, Alonso Hearn M. *Mycobacterium avium* subspecies *paratuberculosis* isolates from sheep and goats show reduced persistence in bovine macrophages than cattle, bison, deer and wild boar strains regardless of genotype. Vet Microbiol 2013;163: 325–334. 10.1016/j.vetmic.2012.12.042 23415474

[7] Abendaño N, Tyukalova L, Barandika JF, Balseiro A, Sevilla IA, Garrido JM, et al *Mycobacterium avium* subsp. *paratuberculosis* isolates induce in Vitro granuloma formation and show successful survival phenotype, common anti-inflammatory and antiapoptotic responses within ovine macrophages regardless of genotype or host of origin. PLoS One 2014;9: E104238 10.1371/journal.pone.0104238 25111300 PMC4128652

[8] Delves PJ, Roitt IV. Roitt's Essential Immunology. Ed. Hoboken, NJ: Wiley-Blackwell ed; 2011.

[9] Good M, Duignan A, Maher P, O´Keeffe J. Veterinary Handbook for Herd Management in the Bovine tb Eradication Programme. Department of Agriculture, Fisheries and Food 2010 Available: http://www.bovinetb.info/docs/veterinary-handbook-for-herd-management-in-the-bovine-tb-eradication-programme.pdf

[10] De La Fuente J., Gortázar C, Juste R. Complement component 3: a new paradigm in tuberculosis vaccine. Expert Review of Vaccines 2016;15: 275–77. 10.1586/14760584.2016.1125294 26605515

[11] Naranjo V, Höfle U, Vicente J, Martín MP, Ruiz-Fons F, Gortazar C, et al Genes differentially expressed in oropharyngeal tonsils and mandibular lymph nodes of tuberculous and nontuberculous European wild boars naturally exposed to *Mycobacterium bovis*. FEMS Immunol Med Microbiol 2006;46: 298–312. 10.1111/j.1574-695X.2005.00035.x 16487312

[12] Roth O, Kurtz J. Phagocytosis mediates specificity in the immune defence of an invertebrate, the woodlouse *Porcellio scaber* (Crustacea: Isopoda). Dev Comp Immunol 2009;33: 1151–1155. 10.1016/j.dci.2009.04.005 19416736

[13] Andersen A., Roth A, Jensen KJ, Erikstrup C, Lisse IM, Whittle H, et al The immunological effect of revaccination with Bacille Calmette-Guérin vaccine at 19 months of age. Vaccine 2013;31: 2137–2144. 10.1016/j.vaccine.2013.02.050 23474315

[14] Hollm-Delgado MG, Stuart EA, Black RE. Acute lower respiratory infection among bacille Calmette-Guerin (BCG)-vaccinated children. Pediatrics 2014;133: e73 10.1542/peds.2013-2218 24379224

[15] Billau A, Matthys P. Modes of action of Freund's adjuvants in experimental models of autoimmune diseases. J Leukoc Biol 2011;70: 849–860. 11739546

[16] Comas I, Chakravartti J, Small PM, Galagan J, Niemann S, Kremer K, et al Human T cell epitopes of Mycobacterium tuberculosis are evolutionarily hyperconserved. Nat Genet 2010;42: 498–503. 10.1038/ng.590 20495566 PMC2883744

[17] Galagan JE. Genomic insights into tuberculosis. Nat Rev Genet 2015;15: 307–320. 10.1038/nrg3664 24662221

[18] Ritz N, Mui M, Balloc A, Curtis N. 2013. Non-specific effect of Bacille Calmette-Guérin vaccine on the immune response to routine immunisations. Vaccine 2013;31: 3098–3103. 10.1016/j.vaccine.2013.03.059 23583897

[19] Buffen K, Oosting M, Quintin J, Ng A, Kleinnijenhuis J, Kumar V, et al Autophagy controls BCG-induced trained immunity and the response to intravesical BCG therapy for bladder cancer. PLoS Pathogens 2015;10: e1004485. 10.1371/journal.ppat.1004485 PMC4214925 25356988

[20] De la Fuente IM. Elements of the cellular metabolic structure. Front Mol Biosci 2015;2: 16 10.3389/fmolb.2015.00016 25988183 PMC4428431

[21] Saeed S, Quintin J, Kerstens HH, Rao NA, Aghajanirefah A, Matarese F, et al Epigenetic programming of monocyte-to-macrophage differentiation and trained innate immunity. Science 2014; 345: 1251086 10.1126/science.1251086 25258085 PMC4242194

[22] Gordon S. Phagocytosis: An immunobiologic process. Immunity 2016;44: 463–475. 10.1016/j.immuni.2016.02.026 26982354

