# Supplementary material for: Increased Lytic Efficiency of Bovine Macrophages Trained with Killed Mycobacteria
Source: PLoS One. 2016 Nov 7;11(11):e0165607. doi: 10.1371/journal.pone.0165607 (PMC5098821; doi:10.1371/journal.pone.0165607)
Supplement: S1 Table — Individual values at testing days 78 and 186 are shown. (DOCX) [file pone.0165607.s001.docx]

|  | **Uptake** | | **Reduction** | |
| --- | --- | --- | --- | --- |
|  | **r** | **p** | **r** | **p** |
| **IFN-PBS OD** (n=32) | -0.81041 | <0.0001 | 0.00163 | 0.9929 |
| **IFN-AVI OD** (n=32) | -0.41097 | 0.0195 | -0.25999 | 0.1507 |
| **IFN-BOV OD** (n=32) | -0.38495 | 0.0296 | 0.04514 | 0.8062 |
| **ELISA OD** (n=32) | -0.74612 | <0.0001 | 0.03395 | 0.8536 |
| **TGF-β** (n=9) | -0.62873 | 0.0697 | -0.21243 | 0.5832 |
| **TNF** (n=9) | 0.83606 | 0.0050 | 0.02235 | 0.9545 |
| **C3** (n=6) | -0.40078 | 0.4310 | 0.78231 | 0.0659 |
