# Supplementary material for: Increased Lytic Efficiency of Bovine Macrophages Trained with Killed Mycobacteria
Source: PLoS One. 2016 Nov 7;11(11):e0165607. doi: 10.1371/journal.pone.0165607 (PMC5098821; doi:10.1371/journal.pone.0165607)
Supplement: S2 Table — MycRed: Least square (LS) mean and standard error of the mean (SEM) mycobacterial count reduction from 2 h post-inoculation to 7 days post-inoculation. ELISA186: LS mean and SEM antibody ELISA OD at 186 days post-inoculation. IFN186: LS mean and SEM interferon release assay at 186 days post-inoculation. ST81: LS mean and SEM skin test thickness increase at 81 days post-inoculation. IL1: LS mean and SEM interleukin 1 gene expression. TGFB: LS mean and SEM transforming growth factor beta gene expression. BCL2: LS mean and SEM bovine B-cell lymphoma/leukemia-2 apoptosis suppressant gene expression. TNF: LS mean and SEM tumour necrosis factor alpha gene expression. C3: LS mean and SEM complement 3 gene expression. Control: Untreated control group. MdR-FD: Killed M. bovis full-dose group. MdR-RD: Killed M. bovis reduced-dose group. (DOCX) [file pone.0165607.s002.docx]

| **Treatment** |  | **MycRed186** | **ELISA186** | **IFN186** | **ST81** | **IL1** | **TGFB** | **BCL2** | **TNF** | **C3** |
| --- | --- | --- | --- | --- | --- | --- | --- | --- | --- | --- |
| Control | Mean | -0.138 | 0.223 | 0.365 | 0.000 | 1.840 | 1.339 | 0.866 | 0.629 | 0.601 |
|  | *SEM* | *0.152* | *0.055* | *0.316* | *0.768* | *0.992* | *0.366* | *0.436* | *0.188* | *0.276* |
| MdR-FD | Mean | 0.834 | 0.372 | 1.445 | 4.650 | 2.320 | 1.278 | 1.175 | 0.388 | 7.301 |
|  | *SEM* | *0.099* | *0.034* | *0.193* | *0.543* | *1.215* | *0.366* | *0.436* | *0.188* | *0.338* |
| MdR-RD | Mean | 0.880 | 0.243 | 0.141 | 0.583 | 4.145 | 0.643 | 1.287 | 0.968 | 2.680 |
|  | *SEM* | *0.107* | *0.036* | *0.207* | *0.572* | *1.215* | *0.366* | *0.436* | *0.188* | *0.478* |

| Difference p-values | | | | | | | | | | |
| --- | --- | --- | --- | --- | --- | --- | --- | --- | --- | --- |
| Control | MdR-FD | **<0.0001** | **0.0005** | **<0.0001** | **<0.0001** | 0.7748 | 0.9097 | 0.6340 | 0.3984 | **0.0006** |
|  | MdR-RD | **<0.0001** | 0.3976 | 0.7641 | 0.6482 | 0.2156 | 0.2267 | 0.5196 | 0.2499 | **0.0327** |
| MdR-FD | MdR-RD | 0.4595 | **0.0010** | **<0.0001** | **<0.0001** | 0.3481 | 0.2652 | 0.8612 | 0.0718 | **0.0042** |
